# Draft Genome Sequences of *Streptomyces scabiei* S58, *Streptomyces turgidiscabies* T45, and *Streptomyces acidiscabies* a10, the Pathogens of Potato Common Scab, Isolated in Japan

**DOI:** 10.1128/genomeA.00062-16

**Published:** 2016-03-03

**Authors:** Tsuyoshi Tomihama, Yatsuka Nishi, Masao Sakai, Makoto Ikenaga, Takashi Okubo, Seishi Ikeda

**Affiliations:** a Laboratory of Plant Pathology and Entomology, Kagoshima Prefectural Institute for Agricultural Development, Kagoshima, Japan; b Faculty of Agriculture, Kagoshima University, Kagoshima, Japan; c Environmental Biofunction Division, National Institute for Agro-Environmental Sciences, Ibaraki, Japan; d Agricultural Research Center for Hokkaido, National Agriculture and Food Research Organization, Hokkaido, Japan

## Abstract

The draft genome sequences of the three pathogens of potato common scab, *Streptomyces scabiei* S58, *Streptomyces turgidiscabies* T45, and *Streptomyces acidiscabies* a10, isolated in Japan, are presented here. The genome size of each strain is >10 Mb, and the three pathogenic strains share genes located in a pathogenicity island previously described in other pathogenic *Streptomyces* species.

## GENOME ANNOUNCEMENT

Potato common scab (PCS) disease, caused by pathogenic *Streptomyces* spp., occurs throughout potato-growing areas in Japan and elsewhere in the world (1). Pathogenic *Streptomyces* spp. produce a virulent phytotoxin, thaxtomin A, a cellulose synthesis inhibitor defective in plant cell walls, and they possess a conserved biosynthetic operon for the synthesis of thaxtomin in a mobile large pathogenic island (PAI) (2–4). In Japan, at least three pathogenic *Streptomyces* spp. have been shown to cause PCS: *S. scabiei*, *S. turgidiscabies*, and *S. acidiscabies* (5). Of these species, both *S. scabiei* (6) and *S. turgidiscabies* (7) are common pathogens in most potato cultivation areas in Japan (8), and reducing the soil pH to <5.2 has been recommended for suppressing PCS (9). However, *S. acidiscabies*, which is an emergent pathogen as a result of a recent acquisition of a PAI, is able to tolerate a lower pH than *S. scabiei* and *S. turgidiscabies*, and there are concerns of its spread in potato-growing areas with low-pH soils (5, 10). While genome sequences are available for several pathogenic *Streptomyces* species (2–4), no genome sequence is available for the Japanese isolates. Here, we present the draft genome sequences of three pathogenic strains isolated in Japan, *S. scabiei* S58, *S. turgidiscabies* T45, and *S. acidiscabies* a10.

The genome sequences of the three pathogenic strains were obtained by assembly of data sets generated by MiSeq paired-end sequence strategies utilizing the SPAdes genome assembler (version 3.5.0) (11). The Prokka annotation pipeline (version 1.11) was used to predict coding sequences (CDSs), rRNA genes, tRNA genes, and noncoding RNA (12). The sequence characteristics of the strains are listed in Table 1. The CDSs obtained from the three sequenced genomes were clustered using the CD-HIT algorithm (13), with a 70% sequence identity cutoff. A total of 18,502 clusters were identified, and of these, 3,080 clusters (16.6%), which included the previously described thaxtomin synthetic genes (2–4), were shared in all strains. These sequences provide a wealth of data for genome comparisons between strains with different pH tolerances, and they enable a great understanding of the emergence of pathogens.

**TABLE 1:** Summary of genome sequencing results in the present study

Strain	Genome size (bp)	G+C content (%)	No. of scaffolds	N_50_ (bp)	No. of CDSs^*a*^	No. of tRNAs	Accession no.
*S. scabiei* S58	10,003,030	71.5	158	193,185	8,629	84	BCMM00000000
*S. turgidiscabies* T45	10,579,795	69.9	103	264,425	9,264	84	BCMN00000000
*S. acidiscabies* a10	10,726,382	70.6	285	82,196	9,295	90	BCMK00000000

a CDS, coding sequence.

### Nucleotide sequence accession numbers.

The nucleotide sequence accession numbers for GenBank are found in Table 1. The versions described in this paper are the first versions.
